# Suicide Risk Assessments Understood as Medical Rituals: Functions and Implications from Societal and Medico-Ethical Perspectives

**DOI:** 10.1007/s11673-024-10419-y

**Published:** 2025-06-03

**Authors:** Antoinette Lundahl

**Affiliations:** Stockholm Centre for Healthcare Ethics, Department of Learning, Informatics, Management and Ethics, Karolinska Institutet, Stockholm, Sweden

**Keywords:** Suicide risk assessments, Suicide prevention, Medical ethics, Rituals

## Abstract

The use of suicide risk assessments in individual psychiatric treatment is widespread and, in many countries, mandatory. However, these assessments exhibit poor predictive accuracy and offer limited clinical value. This raises the question of whether non-medical reasons underpin their continued use. In this paper, suicide risk assessments are interpreted as medical rituals—formalized, repetitive behaviours imbued with symbolic significance that fulfil social functions. Several such functions are proposed, including uniting care providers around shared values in suicide prevention, fostering a sense of safety and control over suicidal behaviour, projecting accountability, and signalling to the public that action is being taken. However, this practice may inadvertently lead to an increase in non-beneficial compulsory admissions, flawed prioritization of patients, and the proliferation of defensive medicine. While the ritualistic use of suicide risk assessments may serve important societal purposes, their potential to harm individual patients renders them indefensible from a medico-ethical standpoint.Instead, evidence-based suicide preventive interventions are recommended. These include implementing general safety measures, equipping psychiatric patients with safety plans, and providing effective mental health treatment according to medical needs.

## Introduction

In many countries, suicide risk assessments, or similar tools, are mandatory for psychiatrists (The National Board of Health and Welfare n.d.; Smith 2022; NICE Guideline NG225 2022). Despite empirical evidence to the contrary, it is widely believed that these assessments help prevent suicides (The National Board of Health and Welfare n.d.; Graney et al. 2020; Smith 2022). Although extensive research has been conducted on suicide prediction, no current assessment tool has demonstrated sufficient accuracy to make its predictions clinically relevant for individual patients (Undrill 2011; Large 2018; Smith 2022). In fact, it has been argued that these assessments may lead to misguided prioritization of patients, increase the use of non-beneficial compulsory admissions, and, as a result, potentially raise the risk of suicide for certain individuals (Walsh et al. 2015; Wang and Colucci 2017; Large 2018; Large and Kapur 2018; Borecky et al. 2019; Smith 2022; Lundahl 2024). Consequently, the mandatory use of suicide risk assessments cannot be considered evidence-based. Other factors must explain their continued widespread use as well as the belief that they do work when they obviously do not.

This paper will contend that these assessments can be compared with rituals. Although rituals lack a universally accepted descriptive definition, they are commonly stipulatively characterized as formalized, repetitive behaviours that hold symbolic or instrumental value and often serve a socially beneficial purpose. For example, they may contribute to social order and meaning, confer privileged status on certain practices, enhance feelings of control and security, and foster a sense of community. These symbolic acts may also be employed with the aim of producing empirical effects, mainly through psychological or social mechanisms (Bell 1992; Stephenson 2015; Arnold et al. 2020).

Drawing on both literature on rituals and empirical data, I hypothesize that these rituals serve several non-medical functions in psychiatric care. This paper will present both the positive and negative aspects of using suicide risk assessments in psychiatric care. The paper will conclude with deliberations on whether this ritualized practice is defensible, considering both medico-ethical and social perspectives.

## The Reasons Why Suicide Risk Assessments Can Be Viewed as Medical Rituals

### Rituals in Medicine

Rituals are prevalent in medicine, though they remain under-researched—perhaps because Western medicine tends to distance itself from symbolic practices that lack a medical evidence base (Stephenson 2015; Arnold et al. 2020). Rituals can be found in various aspects of medical practice, including dress codes and the formalized structure of patient examinations and medical procedures. On the positive side, rituals can enhance efficiency and safety, maintain order, and bring about placebo effects. However, on the negative side, some rituals can perpetuate meaningless or even harmful practices—such as interventions lacking medical value or harming the patients—and obstruct progress and change. Examples of criticized medical rituals are the performance of female circumcision to meet cultural demands and the over-prescription of antibiotics to please patients (Arnold et al. 2020).

Given the influence of rituals in medicine, Arnold et al. (2020) have argued that practitioners should critically reflect on their rituals—considering whether they are constructive and whether they should be maintained, modified, or abandoned. We shall return to this question at the end of this paper.

### The Practice and Empirical Support of Suicide Risk Assessments

Suicide risk assessments, whether based on clinical judgments or structured tools, have low positive predictive value, meaning that only a small fraction of patients identified as high risk will actually die by suicide (Large 2018; Whiting and Fazel 2019; Graney et al. 2020; Fazel and Runeson 2020; Bryan 2021; Bjureberg et al. 2022). Meta-analyses show that nearly half of patients who later die by suicide had previously been classified as low risk, while up to 99.8 per cent of patients deemed high risk will not die by suicide in the near future, and 95 per cent will never die by suicide (Large 2018; Wang and Colucci 2017; Large 2018; Bjureberg et al. 2022).

The low positive predictive value of suicide risk assessments has prompted revised clinical guidelines in countries such as England, Australia, and New Zealand, where clinicians are now advised against using these assessments for suicide prediction and clinical guidance (Graney et al. 2020). In England, however, suicide risk is still expected to be acknowledged in the patient’s treatment plan through “risk formulations” based on historical data, the patient’s strengths and limitations, and current status—equating the factors underpinning traditional risk assessment tools (NICE Guideline NG225 2022). This ambiguity in clinical directives may account for why, despite the updated guidelines, suicide risk assessments continue to be widely practised in the United Kingdom and remain integral to clinical decision-making (Graney et al. 2020; NICE Guideline NG225 2022; Smith 2022). In many other countries, including the United States, the use of these assessments is still mandatory, and failure to employ them can result in litigation or criticism from regulatory authorities (The National Board of Health and Welfare n.d.; Borecky et al. 2019; Graney et al. 2020; Fazel and Runeson 2020).

Even if suicide risk could be assessed with greater accuracy, there are currently few individually targeted interventions that reliably reduce this risk, and none of these interventions are based on assessed suicide risk. For patients with specific diagnoses, pharmacological treatments such as lithium or clozapine have been shown to reduce suicidal behaviour, as have certain forms of psychotherapy (Chiles 2018; Large 2018; Bryan 2021; Smith 2022; Huang et al. 2022) Hospitalization, one of the most common suicide-preventive interventions, has not been demonstrated to reduce suicide risk and may, in fact, increase it for some patients (Large et al. 2014; Borecky et al. 2019; Lundahl 2024). Overall, suicide-prevention measures appear to be most effective when implemented more broadly. For instance, this may involve restricting access to means of suicide within both healthcare settings and society at large or offering crisis support to all patients who might benefit. (Fazel and Runeson 2020; Bryan 2021; Soper et al. 2022).

### The Reasons Why Suicide Risk Assessments Can Be Interpreted as Medical Rituals

Several parallels can be drawn between recognized medical rituals and suicide risk assessments. Firstly, the use of suicide risk assessments as predictive and decision-guiding tools lacks empirical support and may even cause harm (Undrill 2011; Wang and Colucci 2017; Large 2018; Smith 2022; NICE guideline NG225 2022). Their continued prevalence in clinical practice, despite the absence of supporting evidence, would be puzzling if not for the possibility that these assessments serve purposes beyond evidence-based medicine. Secondly, they encourage formalized, stereotypical behaviours; the assessments (or “risk formulations”) follow a predetermined pattern that is repeated at each patient encounter in psychiatry and documented according to local routines (The National Board of Health and Welfare n.d.; Kunskapsstodforvardgivare.se n.d.; NICE Guideline NG225 2022). Thirdly, they fulfil social functions, such as fostering feelings of control and safety, and demonstrating that psychiatry is actively engaged in suicide prevention—“doing something” is often deemed preferable to “doing nothing,” even when the intervention lacks evidential support (Arnold et al. 2020; Graney et al. 2020; Smith 2022).

In this paper, I contend that these parallels justify viewing suicide risk assessments as medical rituals, serving a socially useful function distinct from evidence-based practice. I will further explore these rituals’ potential roles and associated drawbacks, drawing on relevant literature.

## Possible Functions of Ritualizing Suicide Risk Assessment in Psychiatry

### Reducing Suicides by Uniting People Around Shared Values

The “suicide zero” movement, akin to the “war on cancer,” exemplifies how a shared set of moral values can create a sense of community around a common goal—in this case, reducing suicide rates by uniting people over values such as “preventing suicide is a duty” (Stephenson 2015; Smith 2022; Suicidezero.se 2024).

The movement operates on several premises: that suicide is primarily caused by mental illness, that individual suicidality can be accurately predicted and treated by mental healthcare, and that with effective intervention, suicides can be reduced towards zero. This policy has been implemented in many countries, and a central tenet is the integration of suicide risk assessments into standard psychiatric practice (Smith 2022; Boggs et al. 2024). By ritualizing the use of suicide risk assessments, the practice gains a privileged status within the movement (Bell 1992), reinforcing its significance while embedding it within a broader, meaningful narrative.

However, the policy has faced widespread criticism due to its underlying assumptions. Recent research indicates that the link between mental illness and suicide is weaker than previously believed, that individual suicidality is rarely accurately predicted, and that few individual interventions have proven effective in reducing suicide risk (as outlined above). Consequently, the goal of eliminating all suicides is considered unrealistic. Critics also argue that this approach may lead to an overuse of coercive measures and a misallocation of resources (Franklin et al. 2017; Karlsson et al. 2018; Fazel and Runeson 2020; Bryan 2021; Smith 2022).

On the other hand, the movement sends a powerful message of unified communal and governmental commitment to suicide prevention, which can galvanize mental healthcare workers and show the public that healthcare is taking concrete action. Furthermore, by believing in this policy, there seems to be an idea that the goal can be attained, akin to the saying “faith can move mountains.” The latter exemplifies the idea that symbolic acts can produce empirical effects (through indirect, psycho-social mechanisms) (Stephenson 2015; Karlsson et al. 2018).

### Demonstrating Accountability and Upholding Public Trust

When a person dies by suicide, psychiatry is often held responsible for failing to predict and prevent the event, even through coercive measures if deemed appropriate (Catino 2009; Borecky et al. 2019). In response, regulatory bodies typically impose increasingly detailed requirements on healthcare providers, such as mandating additional checklists, protocols, and suicide risk assessments. The completion of these tasks becomes a focal point in the audit process following a suicide (The National Board of Health and Welfare n.d.; O’Neill 2002; Roberts et al. 2008; Fröding et al. 2021; NICE Guideline NG225 2022). However, despite the widespread implementation of these measures, evidence suggests that they do not reduce suicide rates (Undrill 2011; Fröding et al. 2021; Smith 2022).

This increased focus on procedural conformity and administrative control reflects a broader pattern in which authorities adopt ritualized measures to demonstrate accountability and mitigate feared outcomes (O’Neill 2002; Waring and Bishop 2013). This phenomenon has been noted by several scholars, although from different perspectives. Taken together, they describe how, for example, ritualistic assessments, detailed documentation, and risk predictions, are frequently used to exert control over undesirable behaviours and to convey trustworthiness (Foucault 1977; O’Neill 2002; Nowotny 2021). Similarly, by ritualizing suicide risk assessments, authorities can demonstrate their commitment to suicide prevention, confer elevated status on the practice (Bell 1992), and assert control over healthcare procedures, while also sustaining public trust in the healthcare system.

On the other hand, although the intention behind these measures is to enhance the quality of care and public trust in healthcare, there is little evidence to suggest that this form of accountability has successfully increased public trust in healthcare (O’Neill 2002). In fact, one might argue that perpetuating the false belief that suicide risk assessments can prevent suicides could diminish public trust, as the promised outcomes are not realized.

## Arguments Held Against Suicide Risk Assessments in Individual Treatment

### An Increase in Compulsory Admissions

When risk stratifications are conducted, whether referred to as suicide risk assessments or risk formulations, a likely outcome is that they prompt some form of clinical action (Graney et al. 2020; Smith 2022). Although guidelines in some countries advise against using risk assessments for clinical decision-making (NICE guideline NG225 2022), cognitive biases such as availability (the tendency to base decisions on information that readily comes to mind) and anchoring (the tendency to rely too heavily on the initial piece of information presented), alongside the awareness of a potentially threatening outcome, may explain why clinicians appear to disregard this recommendation (Kahneman 2013; Graney et al. 2020; Smith 2022).

A common response when patients are assessed as being at high risk for suicide is to admit them to hospital—through coercive means if deemed appropriate (Wang and Colucci 2017; Borecky et al. 2019; Smith 2022; Lundahl 2024). This approach is based on the assumption that hospitalization can prevent suicide. However, evidence not only suggests that hospitalization often fails to achieve this but also indicates a potential causal link between hospitalization—particularly compulsory admissions—and suicide, to some extent (Large et al. 2014; Walsh et al. 2015; Kapur et al. 2015; Huber et al. 2016; Franklin et al. 2017; Large and Kapur 2018; Jordan and McNiel 2020; Lundahl 2024). Depriving individuals of their autonomy, whether through imprisonment or hospitalization, appears to have suicidogenic effects to some degree (Chiles et al. 2018; Borecky et al. 2019; Smith 2022).

It has been argued that suicide risk assessments may also increase the use of hard paternalism since patients assessed with high suicide risk do not necessarily suffer from decision incompetence in matters of treatment (Owen et al. 2008; Beauchamp and Childress 2019; Bryan 2021; Smith 2022; Lundahl 2024; Lundahl et al. 2024). In this context, hard paternalism refers to the coercion of a decision-competent patient for their own benefit. Such competence should entitle the patient to refuse the treatment offered, as failing to respect this right undermines their autonomy (Beauchamp and Childress 2019).

Taken together, performing risk assessments in psychiatry tends to shift focus from prioritizing what is best for the patient to avoiding calculated risks, potentially through coercive measures.

### An Increase in Defensive Medicine

In this section, I will explore another dimension of risk-averse practices in psychiatry: defensive medicine. When clinicians feel threatened by potential complaints or litigation over outcomes beyond their control, they tend to adopt defensive strategies (O’Neill 2002; Studdert et al. 2005; Kahneman 2013). Defensive medicine refers to medical decisions driven primarily by the need to protect the clinician from legal action, rather than to benefit the patient. This practice can involve unnecessary tests, referrals, documentation, hospital admissions, and coercive interventions, as well as overdiagnosis and the avoidance of patients with complex problems (Studdert et al. 2005; Reuveni et al. 2017; Maughan and James 2017; Graney et al. 2020; Lundahl et al. 2024).

Research indicates that defensive medicine is a widespread issue in both general medicine and psychiatry, leading to the misallocation of resources and potentially exposing patients to futile or even harmful interventions (Studdert et al. 2005; Maughan and James 2017; Reuveni et al. 2017). For example, conducting and accurately documenting suicide risk assessments is widely regarded as time-consuming, even though the practice has limited clinical utility in predicting or preventing suicide (Large 2018; Graney et al. 2020; Fröding et al. 2021). Also, it can feel overwhelming for clinicians to be imposed with the nearly impossible task of predicting and preventing patient suicides on an individual level (Smith 2022). To manage the anxiety and fear associated with this responsibility, clinicians tend to resort to defensive practices, which not only escalate costs and take time but also increase the likelihood of non-beneficial coercive measures (Undrill 2011; Reuveni et al. 2017; Graney et al. 2020; Smith 2022; Lundahl et al. 2024).

## Discussion

In this paper, I have argued that suicide risk assessments can be likened to medical rituals that serve various non-medical functions. Both positive and negative outcomes of these assessments have been suggested. The central question is whether the potential benefits outweigh the drawbacks, including the risk of undermining the patient’s right to autonomy (Beauchamp and Childress 2019). The response to this question largely depends on the perspective adopted—whether the emphasis is placed on the best interests of the individual patient or the broader concerns of society and its governing institutions. Within the field of medical ethics, it is widely accepted that the patient’s best interest should take precedence in clinical decision-making (Beauchamp and Childress 2019). However, the value of societal interests should not be disregarded, hence they will also be addressed.

### Medico-Ethical Deliberations on Upholding Suicide Risk Assessments in Psychiatry

As discussed above, there are several potential positive social functions of these so-called medical rituals. However, none of these functions serves the patient’s best interest, which is a fundamental principle of medical ethics (Beauchamp and Childress 2019).

There have been proposals to modify the use of suicide risk assessments to better serve individual patients. One suggestion involves collaboratively exploring the patient’s unique needs and suicide risk factors to make the assessment process more therapeutic (Hawton et al. 2022). While this approach may appear more appealing than current practices, it remains grounded in risk factors with limited predictive value and may lead to the same negative consequences as conventional suicide risk assessments (Large et al. 2022). Engaging in discussions with patients about managing crises and suicidal impulses does not necessitate a formal risk assessment and should, arguably, be offered to all individuals experiencing mental distress, regardless of their assessed risk level (Soper et al. 2022).

Other proposals have been to enhance the predictive accuracy of the current risk assessments through real-time monitoring or machine-learning techniques. However, these methods are, just as the current assessments, likely to exhibit a low positive predictive value due to the rarity of suicide, its low incidence within short time frames, and the fact that it is often precipitated by multiple, dynamic factors (Large 2018). Reviews of computer-assisted risk estimations support this conclusion (Whiting and Fazel 2019), casting doubt on their ability to enhance clinical decision-making compared to current practices. On the other hand, even a low positive predictive value could be considered useful if it led to beneficial and non-harmful interventions. As argued in this paper, this is not always the case.

Additional proposals advocate leveraging the negative predictive value of risk assessments—defined as the proportion of patients correctly identified as low-risk—to exclude such patients from further interventions, with the aim of making care more cost-effective (Whiting and Fazel 2019; Seyedsalehi and Fazel 2024). This approach, however, is also problematic since approximately half of those who later die by suicide are likely to be assessed in the lower-risk categories (Large 2018). Furthermore, while cohorts with severe mental disorders demonstrate higher negative predictive value (Whiting and Fazel 2019), deprioritizing these individuals—who could substantially benefit from psychiatric care—merely because they are not deemed suicidal appears neither reasonable nor ethically defensible.

In summary, despite ongoing research into suicide risk assessments, the fundamental issues of low predictive accuracy and limited clinical utility are unlikely to be resolved in the foreseeable future. Continuing with the use of suicide risk assessment just because they may one day become beneficial is not a compelling justification—particularly when the practice carries negative consequences for patients.

The potential secondary harms of making suicide risk assessments, such as more compulsory admissions, misguided prioritization, and the practice of defensive medicine raise significant ethical concerns. Unlike the proposed benefits, these outcomes can seriously affect individual patients. Firstly, patients are subjected to screening-like assessments that may lead to compulsory admission, even when they do not benefit from it or are decision-competent to refuse treatment (Borecky et al. 2019; Beachamp and Childress 2019; Smith 2022; Lundahl 2024). Secondly, patients may be wrongly prioritized, missing out on beneficial treatments because clinical decisions rely too heavily on risk assessments rather than the patient’s actual medical needs (Large 2018; Smith 2022). Thirdly, psychiatric care may become less accessible, with patients subjected to unnecessary examinations and interventions as doctors prioritize litigation-risk avoidance over evidence-based care (Catino 2009; Reuveni et al. 2017).

In conclusion, when weighing the potential benefits and harms, it is difficult to argue that the continued ritualistic use of suicide risk assessments is ethically defensible when prioritizing the patient’s best interest.

### Social Reasons for Upholding Suicide Risk Assessments in Psychiatry

For society as a whole, feelings of trust, security, and control are typically desired (Foucault 1977; O’Neill 2002). When control over individuals proves difficult—such as in the case of suicides—an illusion of control can serve as a useful substitute (Nowotny 2021). The potential infringement on individual autonomy, through the implementation of extensive control measures, is often considered a secondary concern. As Nowotny (2021) observes:

Rare are the moments in history when, given a choice between security and freedom, people chose the latter. Security and surveillance can always be sold as being for our own good, while the idea of freedom remains elusive.

Thus, maintaining the practice of suicide risk assessments, despite their failure as predictive tools, serves a societal purpose by fostering a sense of safety.

Another important social consideration is that doctors seek to protect themselves from litigation. Proving that a sound clinical decision was made before a suicide can be challenging, particularly because decisions are often judged based on their outcomes. Demonstrating adherence to established protocols and employing risk assessment tools—which are seen as more objective than clinical judgment—provides a more defensible position (Hacking 1990; O’Neill 2002; Roberts et al. 2008; Kahneman 2013; Nowotny 2021).

Rituals that unite people around shared beliefs to achieve empirical ends can also be of societal importance. As previously noted, the suicide zero movement, which advocates for the practice of suicide risk assessments to reduce suicides, exemplifies this phenomenon. In this context, the practice can provide a sense of meaning, community, and empowerment in the collective effort to prevent suicide (Stephenson 2015; Arnold et al. 2020; Smith 2022).

### What Should Be Done? Some Remarks

Returning to the question posed by Arnold et al. (2020): should this medical ritual (of conducting individual suicide risk assessments) be maintained, modified, or abandoned? From a medico-ethical and clinical standpoint, I argue that it should not be maintained. While these assessments were likely introduced with the intention of aiding suicide prevention, it is now broadly acknowledged that they fail to fulfil this purpose (Large 2018; Graney et al. 2020; Fazel and Runeson 2020; Smith 2022). However, human behaviour is not always driven by rationality, and rituals can hold significant social value, as discussed throughout this paper. While readers may have differing opinions, I maintain that the patient’s best interest should take precedence over societal concerns. This would support discontinuing the use of suicide risk predictions in individual patient treatment. Holding that view does not mean clinicians should not address the patient’s suicidal thoughts and behaviours when appropriate—I argue quite the contrary.

Nevertheless, for both the public and the mental healthcare system to accept the abandonment of suicide risk assessments, the rationale must be clearly explained, and alternative approaches to managing suicidality be offered. Without this, current practices may be viewed as “better than nothing” in addressing suicidal patients (Graney et al. 2020).

One challenge in providing the necessary explanations is the general difficulty people, including medical professionals, have in understanding statistics (Kahneman 2013; Maughan and James 2017). This can result in overestimations of absolute risk and the predictive value of individual risk assessments. By presenting statistical outcomes in clear, accessible language, with concrete examples, researchers can improve health literacy (Maughan and James 2017) and help clinicians better grasp the value of suicide risk assessments.

### Recommendations on How to Move Forward

Suicide is a chance and rare event, making it challenging to forecast at a clinically relevant individual level. Individuals may die by suicide regardless of whether they have a mental disorder or are assessed as being at high or low risk. Moreover, even if suicide risk assessments were to become more accurate, there are few individual interventions that directly and effectively target suicidality. Crucially, none of these interventions require, nor should they rely on, suicide risk assessments (Smith 2022; Large et al. 2022; Soper et al. 2022). As other researchers have argued, acknowledging this reality is an essential step towards advancing suicide prevention efforts (Soper et al. 2022).

I propose that suicide risk assessments be replaced with evidence-based suicide prevention interventions. One of the most effective strategies is restricting access to lethal means on a community-wide scale, such as by reducing opportunities for jumping from bridges or limiting access to toxic substances and firearms (Fazel and Runeson 2020; Bryan 2021). Other potentially effective interventions for reducing suicidal behaviour include improving access to psychiatric and social treatments and providing safety plans, incorporating coping skills, for all individuals receiving mental healthcare (Nuij et al. 2021; Smith 2022; Soper et al. 2022). Furthermore, essential measures involve supporting patients’ autonomy by encouraging active participation in their care, helping them establish a meaningful life beyond mental healthcare, and minimizing the use of compulsory admissions wherever possible (Bryan 2021; Lundahl 2024).

Overall, delivering psychiatric interventions based on individual needs and anticipated benefits, rather than relying on risk assessments, could enhance the effectiveness, accessibility, and overall benevolence of care. Consequently, suicidality associated with mental illness might be reduced (Smith 2022). Paradoxically, the goal of reducing suicides appears more attainable when we move away from attempts to “control” individual suicidality—such as through risk assessments or compulsory admissions—and instead refocus on psychiatry’s core strength: the treatment of mental disorders.

However, the current practice is unlikely to change without endorsement from governing bodies. Thus, removing the requirement for suicide risk assessments/formulations at the policy level would be a critical first step. As O’Neill (2002) suggests, assessing healthcare quality should be done by individuals with sufficient experience, time, and expertise to evaluate it properly. This would require moving away from focusing on easily quantifiable performance indicators, such as the performance of risk assessments.

## Conclusions

In summary, suicide risk assessments can be understood as medical rituals that serve several important non-medical functions. This perspective helps to explain their entrenched position in psychiatry and might justify their continued use—despite their lack of clinical utility in predicting or preventing suicide—if they were harmless. However, as outlined in this paper, the potential harm these assessments may inflict on individual patients makes them indefensible from a medico-ethical standpoint.

I contend that significant benefits could be realized by phasing out suicide risk assessments and removing the requirement for clinicians to prioritize “risk” in patient management plans or clinical decision-making. Instead, the focus should shift towards empowering patients’ autonomy, improving access to healthcare and social services, investing in general safety measures across society, and delivering mental healthcare based on medical needs rather than perceived risk.

## Data Availability

Not applicable since this is a normative study.
